# Pretreated Butterfly Wings for Tuning the Selective Vapor Sensing

**DOI:** 10.3390/s16091446

**Published:** 2016-09-07

**Authors:** Gábor Piszter, Krisztián Kertész, Zsolt Bálint, László Péter Biró

**Affiliations:** 1 Institute of Technical Physics and Materials Science, Centre for Energy Research, H-1525 Budapest, P.O. Box 49, Hungary; kertesz@mfa.kfki.hu (K.K.); biro@mfa.kfki.hu (L.P.B.); 2 Hungarian Natural History Museum, H-1088 Budapest, Baross utca 13, Hungary; balint@nhmus.hu

**Keywords:** butterfly wing, biomaterial, photonic crystal, vapor sensing, atomic layer deposition, optical spectroscopy

## Abstract

Photonic nanoarchitectures occurring in the scales of Blue butterflies are responsible for their vivid blue wing coloration. These nanoarchitectures are quasi-ordered nanocomposites which are constituted from a chitin matrix with embedded air holes. Therefore, they can act as chemically selective sensors due to their color changes when mixing volatile vapors in the surrounding atmosphere which condensate into the nanoarchitecture through capillary condensation. Using a home-built vapor-mixing setup, the spectral changes caused by the different air + vapor mixtures were efficiently characterized. It was found that the spectral shift is vapor-specific and proportional with the vapor concentration. We showed that the conformal modification of the scale surface by atomic layer deposition and by ethanol pretreatment can significantly alter the optical response and chemical selectivity, which points the way to the efficient production of sensor arrays based on the knowledge obtained through the investigation of modified butterfly wings.

## 1. Introduction

Nowadays, monitoring the air quality of homes, work places, and industrial facilities is becoming more and more important. For the efficient characterization of the ambient atmosphere, gas and vapor sensors are required which combine high sensitivity and chemically selective detection of volatile organic compounds (VOCs) with low power consumption and fast response time, and operate in ambient air. Sensors based on photonic crystal–type nanoarchitectures may offer an optimal solution to this problem due to the fast development of the response signal (color change) and relatively easy optical readout [1,2]. Although mankind discovered and first produced three-dimensional (3D) photonic crystals almost 30 years ago [3], the low-cost and large-scale production of these intricate structures working in the visible spectrum has not yet been achieved. In contrast, nature produced photonic nanoarchitectures in the animal kingdom more than 500 million years ago [4] which exhibit great diversity: from the one-dimensional lamellar multilayer structures [5,6], through the ordered [7] and quasi-ordered [8] inverse opal structures, to the Morpho-type Christmas tree–like structures [9], many types of color-generating nanoarchitectures can be found [10]. From the point of view of a materials scientist, these are cheap and ready-made nanoarchitectures produced at a macroscopic size which can instantly be used in potential applications as prototypes. The information obtained through these experiments can be used effectively to prepare “butterfly-based” bio-inspired photonic nanoarchitectures with desired optical properties that are compatible with the requirements of mass production.

The vapor-induced color changes were investigated in many types of photonic nanoarchitectures occurring in insect species [1,11,12,13,14,15,16,17] which were found to be vapor-selective, and by taking inspiration from these photonic nanoarchitectures, artificial optical vapor sensor materials were recently developed [18,19,20,21].

**Figure 1:**
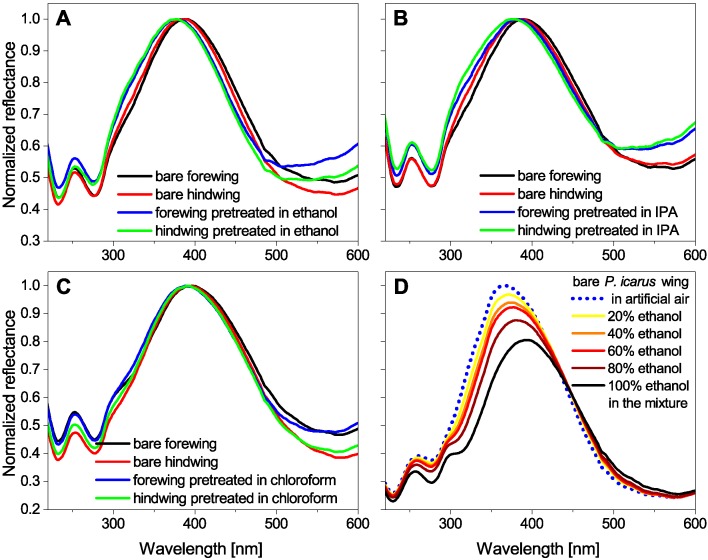
The two-week pretreatment of the *Polyommatus icarus* wings did not alter the structural coloration measured in ambient air as can be seen in case of (**A**) ethanol; (**B**) isopropanol; and (**C**) chloroform, as the measured maximal spectral variation was only 10 nm in the case of ethanol pretreatment. In contrast, (**D**) the color change effect caused when passing vapors of different concentrations over the pristine wing in the measurement cell can be clearly seen as it generated more than 25 nm of spectral shift between the artificial air and the saturated ethanol vapor exposition.

Recently, we explored the VOC-sensing capabilities of the wing scales of nine Lycaenid butterfly species [22]. In the dorsal wing scales of the males of these species, a quasi-ordered three-dimensional photonic nanoarchitecture can be found, which is responsible for the vivid blue coloration [23]; for SEM and TEM images of *P. icarus* wing scales and for the other eight Lycaenid butterflies, see Figure 1 of Reference [23]. These nanoarchitectures, the so-called “pepper-pot” structures, are spatially periodic dielectric nanocomposites constituted of a chitin matrix with embedded air holes [7]. If the characteristic sizes of the building elements of the nanocomposite are in the range of the visible light’s wavelengths, which is typically hundreds of nanometers, and the refractive index contrast is suitable, then structural color in the visible range may be generated. This is analogous with semiconductor crystals, which exhibit a forbidden gap for electrons [24]. Similarly, the photonic crystals possess a forbidden gap for photons of a certain wavelength range (energy) which does not allow the propagation of light through the nanocomposite.

The change of the structural color, i.e., the energy level variation of the forbidden gap in the photonic crystal’s band structure, can be induced in two different ways: by the alteration of the refractive index of one or both of the optically different building materials, or by the change of the characteristic size of the building elements [11,25,26]. It was shown recently that in the case of blue butterfly wing scales possessing pepper-pot–type photonic nanoarchitectures, the color change process depends on the vapor concentration: at lower concentrations the governing process is the refractive index contrast change through the capillary condensation of the vapors, and the change of the characteristic sizes through the swelling of the chitin nanoarchitecture becomes dominant only at higher concentrations [27]. The capillary condensation and the swelling are both based on the interaction of the vapors with the chitin matrix; therefore, both depend on the material properties of the test volatiles and the surface properties of the chitin nanoarchitecture. This results in chemically selective sensing through the chemically selective optical reflectance change which also provides relatively high sensitivity [27].

Both the chemical selectivity and the sensitivity could be improved if arrays of sensitized butterfly wings would be used. A possibility that comes immediately to one’s mind is to use for sensitization one or several of the volatiles which we used for the demonstration of chemical selectivity. As these substances—as shown by their individual trajectories in the concentration–optical response diagrams [27]—clearly have different chemical interactions with the chitin of the photonic nanoarchitecture, so it may be expected that they will modify the chemical properties of the nanoarchitecture. In our current work we show that the chemical sensitivity and selectivity of the butterfly wing–based sensors can be tuned over a wide range by well-chosen pretreatment materials. In particular, the soaking in ethanol increased both the sensitivity and the selectivity of the sensor (especially at the low concentration range), while the pretreatment in chloroform and isopropanol did not result in improvement, but in the loss of chemical selectivity. These findings were compared to our previous results [27] where atomic layer deposition (ALD) was applied for the conformal surface modification of wing-scale nanoarchitectures and the vapor-sensing properties of these modified wings were investigated.

## 2. Materials and Methods

The investigated *Polyommatus icarus* specimens were obtained from the curated Lepidoptera Collection of the Hungarian Natural History Museum. Vapor sensing measurements were carried out fixing the butterfly wing in an air-proof aluminum cell covered with a quartz glass window to provide UV transmittance [26]. During the measurements a constant gas flow of 1000 mL/min through the cell was maintained. The vapor concentration was set by switching digital mass flow controllers (Aalborg DFC, Aalborg Instruments & Controls Inc., Orangeburg, NY, USA) to let pass synthetic air (80% N_2_, 20% O_2_) and saturated volatile vapors from gas bubblers in the required ratio. Experiments were carried out changing the concentration and nature of test vapors while monitoring the spectral variations in time: 20 s mixture flow was followed by 60 s of synthetic air flow, to purge the cell. A 60 s purging was required to recover the initial reflectance value before the introduction of the next vapor mixture. When the vapor mixture was passed through the cell containing the wing a spectral difference appeared at certain wavelengths as compared to the pure air case. For the normal incidence illumination of the samples an Avantes AvaLight DH-S-BAL light source was used and the highest reflected signal was captured at ~45° and were measured with an Avantes HS 1024*122TEC spectrometer (Avantes BV, Apeldoorn, The Netherlands).

The 5-nm-thick Al_2_O_3_ film was grown in a Picosun SUNALE R-100 type ALD reactor (Picosun Ay, Espoo, Finland) to the wings of *P. icarus* butterfly. Electronic grade purity trimethylaluminum and H_2_O were used as precursors. The carrier gas and purging medium was 99.999% purity nitrogen. The pulse and purging lengths were previously optimized so that the precursors would penetrate into the porous structure of the butterfly wing scales and the growing film would cover its inferior walls evenly. Further details in [28].

Pretreated samples were obtained by soaking *P. icarus* butterfly wings in three types of—ethanol, isopropanol, and chloroform (analytical grade, VWR International Ltd, Radnor, PA, USA)—for 14 days to modify the surface of the photonic nanoarchitecture. After the complete drying the standard vapor sensing experiments were carried out to investigate the differences in the selectivity and the sensitivity of the modified wings.

The Principal Component Analysis (PCA) was carried out using Originlab OriginPro 2015 software (OriginLab Co., Northampton, MA, USA).

## 3. Results

The detailed investigation of the vapor-sensing properties of the pretreated *Polyommatus icarus* butterfly wings was preceded by the control of the possible color modification caused by the pretreatment method. The spectral characteristics were investigated using an integrating sphere optical setup [29] and the reflectance spectra of the untreated and the soaked wings were measured. For each treatment we used all four wings of *P. icarus* males, two of which were untreated and two of which were soaked for 14 days. The results of the reflectance measurements can be seen in Figure 1A–C for the three materials.

One can see that the normalized spectra of all four wings (two pretreated vs. two pristine) are highly similar for each of the volatiles used for pretreatment: the maximal difference in the peak wavelength values is 10 nm in the case of the ethanol pretreatment (Figure 1A) and all the other measured spectral differences are lower than that. It clearly shows that the spectral variance of the measured color is in the range of the natural color variability of these insects which is typically ±15 nm using this measurement method [29], meaning that structural changes caused by the volatile pretreatment of the photonic nanoarchitecture can be excluded. Furthermore, this color variability is significantly lower than the spectral shift which is measured during vapor exposition (Figure 1D). The alteration of the surface properties of the pretreated wing-scale nanoarchitectures compared to the untreated wings can be investigated in more detail with vapor-sensing experiments.

**Figure 2:**
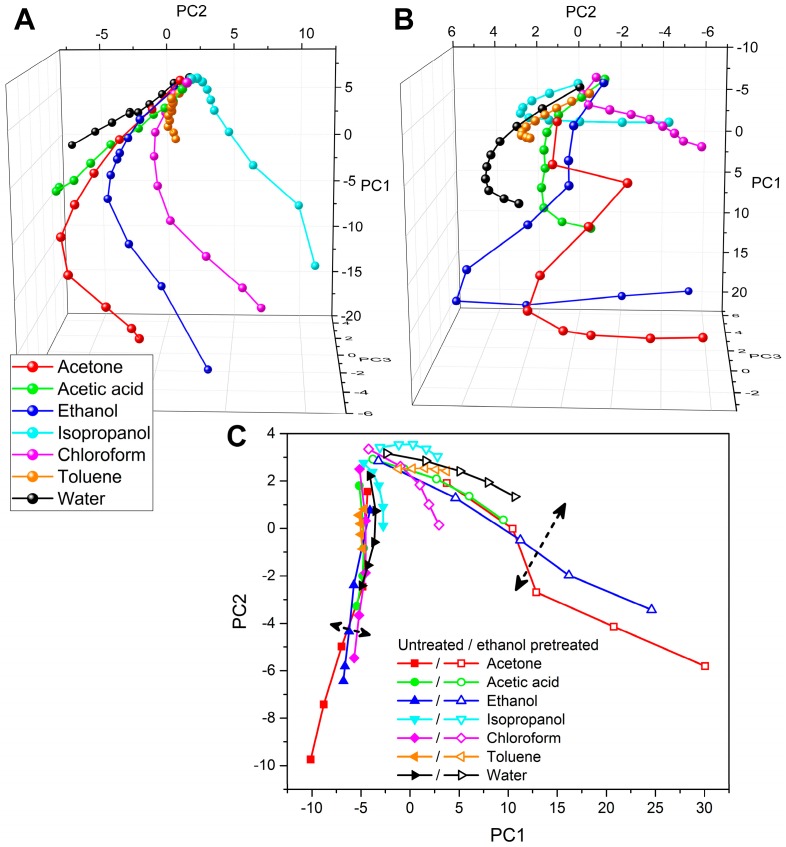
PCA score plots of (**A**) the untreated and (**B**) the 14-day-ethanol-pretreated *Polyommatus icarus* wings were used as a sensor material. The vapor concentration increases from the top to the bottom (to saturated vapors) in both graphs. The low-concentration behavior was investigated in more detail using PCA (**C**). The PCA score plots of the untreated and ethanol-pretreated wings are compared in the low-concentration range where the concentration increases from the top-left corner to a 50% vapor concentration radially (regarding the common origin of the curves as a center), in both cases. The two arrows are a guide for the eye: both show the spatial spread of the vapor-sensing trajectories, which is significantly larger in the case of the ethanol-pretreated sample.

The vapor-sensing experiments were performed using the pristine, ALD-modified, and volatile-pretreated *P. icarus* wings. In every experiment seven test vapors in 10 concentration steps (+10% each) were applied from artificial air (0%) to saturated vapors (100%). In Figure 1D, the typical reflectance spectra of an untreated wing can be seen during the ethanol vapor exposition of different concentrations (only five of them are shown for a clearer view). The typical effect caused by the vapors can be clearly seen in the graph: red-shift and slight desaturation occur and both increase with the vapor concentration. These changes are typical for the applied materials but every volatile induces vapor-specific spectral changes [27]. Earlier we showed with temperature-dependent vapor-sensing experiments that the vapors condensate into the nanoarchitecture through capillary condensation [30] and, due to the liquid layer, the swelling of the chitin nanoarchitecture occurs as well, which enables both the higher-than-expected spectral response [15] and the material-specific behavior during the vapor exposition [15,27]. The obtained spectral responses of the untreated wings were analyzed using PCA and the results were plotted in a three-dimensional (3D) diagram. In Figure 2A the 3D diagram with the PCA score plot (cumulative variance of the principal components (PCs) were 99%; PC1 = 70.92%, PC2 = 19.52%, PC3 = 8.67%) of the seven test vapors can be seen (seven vapors × 10 concentrations = 70 data points). The vapor concentration increases from the top to the bottom of the diagram. The trajectories of the individual vapors are well separated and have individual directions which represent the chemical selectivity of the sensor.

To modify the surface of the photonic nanoarchitecture in the volume of the blue wing scales, two-week-long soaking using three different volatiles (ethanol, isopropanol, and chloroform) was applied. After complete drying, the reflectance spectra of the three types of samples were measured and no alterations in the (dry) color were observed, as can be seen in Figure 1A–C. To investigate the effect of the pretreatment on the vapor-sensing properties, the same experiment described earlier was carried out using the three soaked samples. In the case of isopropanol- and chloroform-pretreated samples, the sensitivity of the wing for the seven test vapors was retained, but the trajectories of the different substances almost overlapped; therefore, the chemical selectivity was mostly lost. The spectral results of the ethanol-pretreated sample showed significant modification when they were analyzed using PCA. The PCA scores (cumulative variance of the PCs > 99%; PC1 = 88.15%, PC2 = 8.31%, PC3 = 3.17%) of the seven vapors using the pretreated wing as a sensor material were plotted together in Figure 2B. The trajectories of the different vapors in Figure 2B are rearranged compared to the untreated wing case (Figure 2A). One may remark that for the untreated sample in the range of low concentration, a certain degree of overlap of the different trajectories may be observed. To compare the low concentration behavior of the untreated and the ethanol-pretreated samples, PCA evaluation of the two data sets containing the <50% concentrations was carried out together. These PCA scores were plotted in Figure 2C. The cumulative variance of the first two PCs was 99% in this case (PC1 = 85.17%, PC2 = 13.38%); therefore, a two-dimensional graph was generated. In Figure 2C, the PCA scores of the untreated wing are noted with filled symbols while the ethanol-pretreated results are noted with hollow symbols. The vapor concentration increases from the top-left corner radially (regarding the common origin of the curves as a center). One can see that the PCA scores of the two samples are differently directed, which shows the changed vapor-sensing behavior of the pretreated wing (in accordance with the findings based on Figure 2A,B), but it is worth noting the different characteristics of the vapor-sensing trajectories of the two samples: in the case of the untreated wing the low-concentration trajectories are close to each other and almost parallel, which denotes moderate chemical selectivity in this sensitivity range, while the vapor-sensing trajectories of the ethanol-pretreated sample have a much broader spread with differently directed branches, indicating that the pretreatment caused the gain of chemical selectivity here.

**Figure 3:**
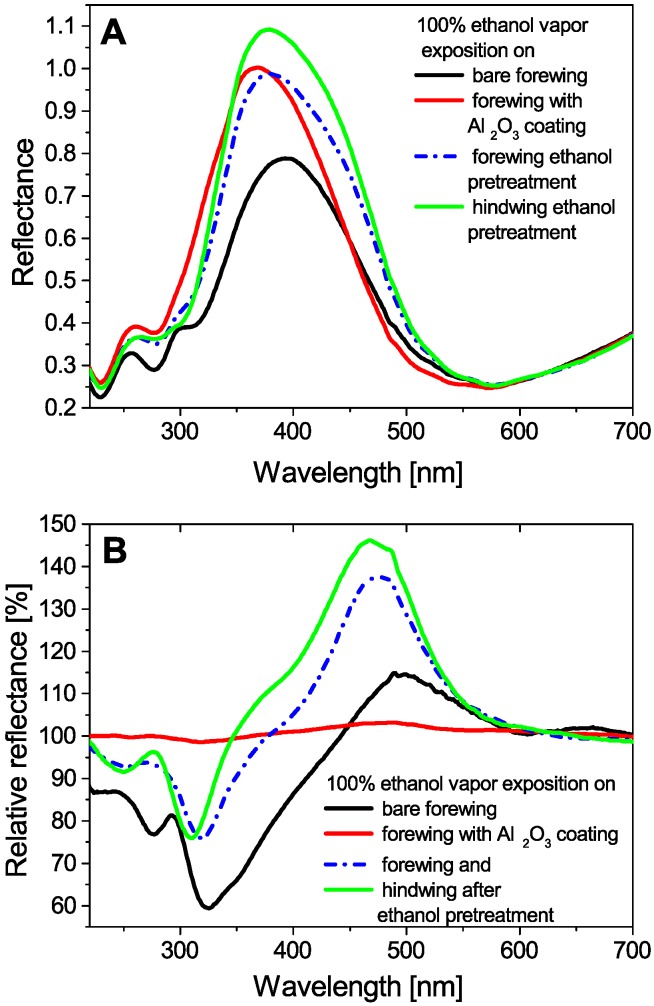
(**A**) Wing reflectance of the bare and the modified samples during saturated (100%) ethanol vapor exposition. (**B**) When the color of the butterfly wing in artificial air is selected as a reference, the resulting relative reflectance curves clearly show spectral change enhancement of the ethanol-pretreated samples: the response signal doubled during saturated ethanol vapor exposition.

It is important to show that in addition to the change in the character of the chemical selectivity, the magnitude of the spectral response increased significantly, especially in the case of the ethanol and acetone test vapors. For these two vapors, one may remark on the pronounced separation of the wetting and swelling phases [27]. To compare the maximal spectral responses during vapor exposition in each experiment, the relative reflectance spectra were introduced, which shows the spectral response of the samples using the *P. icarus* wing reflectance in artificial air flow as a reference (the blue dotted spectrum in Figure 1D). The wing reflectances were plotted in Figure 3A where the Avantes white diffuse standard (WS-2) was used as a reference. The maximal relative reflectance curves, showing the alterations from the butterfly wing in artificial air (initial reflectance), are shown in Figure 3B.

The resulting spectra of the four sensor materials (untreated, Al_2_O_3_-coated, and two ethanol-pretreated) during saturated ethanol vapor flow were plotted in Figure 3A together and the relative reflectance spectra showing maximal spectral responses were plotted in Figure 3B. One can see in Figure 3B that the maximal intensity of the relative reflectance curves is more than doubled when the butterfly wings were pretreated in liquid ethanol for two weeks. This enhanced vapor sensing is reproducible, too: two wings of the same specimen were pretreated under the same conditions and after the measurements, the significant spectral enhancement of both wings was observed. Under identical conditions, the wing conformally covered by Al_2_O_3_, although it exhibited increased/shifted reflectance in artificial air (Figure 3A), completely lost sensitivity during the vapor exposition (Figure 3B).

## 4. Discussion

The structural modification of *Polyommatus icarus* wing-scale nanoarchitectures can cause the color change of the wings: in Reference [28], a slight color shift of the wing reflectance peak in the blue wavelength region was observed when the 5 nm Al_2_O_3_ film was conformally grown on the sample. This is in accordance with our expectations as the 5-nm-thick dielectric coating slightly modifies the pore size of the photonic nanoarchitecture and also the refractive index contrast of the structure, resulting in the shift of the reflectance peak on a small scale. The 14-day soaking of the *P. icarus* wings in three different volatiles can modify the surface properties of the photonic nanoarchitecture as the epicuticle layer covering the scales is mainly constituted of chitin [31]. The unaltered reflectance curves of the pretreated samples in Figure 1A–C clearly show that the materials used for pretreatment did not change the three-dimensional structure of the photonic nanoarchitecture, as the smallest structural change would cause a shift of the structural color as was found in the case of the ALD-coated samples [28]. Furthermore, we showed in the case of nine closely related polyommatine butterfly species that their species-specific photonic nanoarchitectures showed the same characteristics and only minor (but specific) differences were found, but these differences resulted in well-defined species-specific wing colorations [23].

Recently, the vapor-sensing experiment was carried out using the ALD-coated *P. icarus* wing to investigate the effect of a conformal layer of differing chemical composition on the surface of the photonic nanoarchitecture. The results were analyzed using PCA, similar to what is shown here. The PCA score plot of the ALD-coated wing can be seen in Figure 3 in Reference [27] and can be compared with the untreated and the pretreated results. Although the experiment with the ALD-coated sample served to improve the understanding of the interaction between the volatiles and the chitin nanoarchitecture, the significantly reduced spectral response does not support the better practical applications (see the red curve in Figure 3B). On the other hand, the reduced spectral response convincingly shows that not only the structure of the nanoarchitecture is relevant, but the chemical composition of the material from which the nanocomposite is built is also important. In fact, Figure 3B indicates that most of the chemical selectivity arises from the characteristic chemical interaction of chitin with the condensed volatiles, namely the swelling of the chitin nanoarchitecture which was eliminated by applying the ALD coating.

In accordance, the enhancement of the spectral response in the ethanol-pretreated samples (Figure 3B) may be attributed to the modification of the epicuticle surface layer which conformally covers the whole nanoarchitecture and other components of the scale surface [32]. With the long-term treatment of wings in volatiles, the chemical composition of the epicuticle may have changed [33,34,35,36,37] and this caused the alteration of the surface properties as well, which led to the increased sensitivity through increased capillary condensation as can be seen in Figure 3B.

## 5. Conclusions

During the millennia of evolution nature has developed many functional materials which are envied by materials scientists. These often intricate nanomaterials have a decisive significance in the life functions of living beings; therefore, presumably millions of “engineer hours” lie in their proper optimization. The wing coloration of the blue polyommatine butterflies constitutes a sexual communication channel [23] which affects the photonic nanoarchitectures, producing the structural color. We showed in our recent work that the photonic nanoarchitectures occurring in the wing scales of polyommatine butterflies are species-specific and are stable over time; therefore, the structural color they generate only has minor natural variability [29], making them suitable as prototypes of photonic nanoarchitectures with 3D porous nanostructures.

To tune the sensitivity of the sensor material, the modifications of the surface properties of the photonic nanoarchitecture were applied. To achieve this, we pretreated the butterfly wings, soaking them in several kinds of volatiles. This is a cheap way of altering the surface properties of the photonic nanoarchitectures occurring in the scales. At the same time, this procedure can be easily scaled up for practical applications. After the vapor-sensing measurements, the ethanol pretreatment provided the most significant results—more than doubling the maximal spectral response—while the chemical selectivity increased at lower vapor concentrations (<50%) and remained almost unchanged at higher (>50%) concentrations. The treatment of the wings resulted in the functionalization of the epicuticle layer covering the photonic nanoarchitecture, which has become sensitized that way. The marked differences found between the increase in selectivity and sensitivity after soaking in ethanol and the loss of selectivity due to soaking in chloroform and isopropanol clearly indicate that careful choosing of the soaking liquid offers a wide range of tuning options for the optical response of butterfly wings.

These results show how to tune the response of the photonic nanoarchitectures by the modification of their surface. By the sensitization/desensitization of the sensor material for certain volatiles, arrays of sensors can be created, which can significantly enhance both the sensitivity and the chemical selectivity of the system, offering the possibility of “fingerprinting” various volatile substances.

## Abbreviations

VOC	Volatile Organic Compound
ALD	Atomic Layer Deposition
PCA	Principal Component Analysis
